# Professional Etiquette

**Published:** 1863-01

**Authors:** W. H. Atkinson


### PROFESSIONAL ETIQUETTE.

(Read before the N. Y. Society of Dental Surgeons.)

BY W. H. ATKINSON, D. D. S.

ETIQUETTE—Literally a code of manners concisely written, usually upon a court card.

Polite ceremony used between associates to which tacit or expressed assent is given by all.

A strict observance of this code would do away with all contention and injustice among members of communities and professions.

But he who desires to infract some of the requirements of a strict etiquette, for real or imaginary objects, will be most likely to demur to a broad and honest application, of an impartial exercise thereof, and thus inaugurate infraction of its harmonious workings by partial and onesided definitions of its various provisions expressed or implied.

The very prime requirement of a perfect etiquette is to hospitably entertain any and every man's thought, opinion, or belief, and patiently investigate it in the spirit of honest research, that enlightenment may come to all minds, and unmixed truth prevail in the premises.

The need of a system of etiquette implies either ignorance or dishonesty, in the body to which it is proposed to introduce it.

If every individual were absolutely clean in all his purposes, a history of his daily life would be the best possible code of ethical behavior.

But as we are all born in igorance of all things save only our instructive wants, it is next thing to impossible to be clean in our inmost conceptions of the laws of life on society, because we can not distinguish the clean until we have conceived the unclean exercise of the mental, bodily, and social powers.

Each one imbibes his instructions from what to him is a superior source, and hence he thinks he is right in all his conceptions, no matter how narrow or erroneous soever they may be.

The more contracted and completely isolated our views may be, the more distinctly are we willing to pronounce their certainty of rectitude.

As we extend our survey, to the utmost border of the limits of that which we have set down as knowable, we are unconsciously led beyond our prescribed boundary, and startled to find there are yet lengths and depths beyond not dreamed of in our philosophy.

Probably the hardest thing for a bigoted and small mind to encounter is the conception of its own bigotry and insignificence.

Being born into new light is what pains us, because we can no longer adore ourselves for our wonderful attainments, talents, and power.

Probably the spirit most to be desired is a quiet and teachable mind, acute and active, but humble, inquiring, and persistant.

It will ever be in vain to enact laws and make a beautiful record of than on ever so elegant parchment if we fail to imprint and integrate them within our moral and sentient being.

There has been no lack of laws just and equal, (for the obedience of the second and third persons, for whom they were made,) but of integral obedience there has been a general dearth.

Law makers usually become so familiar with the lack of sacredness in themselves that it is no marvel that they should so universally become law breakers when occasion tempts to the infraction, relying for immunity from the penalty, upon their ability at special pleadings, or explaining away the feature obnoxious to their gratifications.

Each earnest searcher for truth can tolerate the presence of strength or weakness in his fellows, well knowing that, whatever his present strength may be, he can well remember when he was all weakness.

Be assured he who is afraid to treat courteously every member of his community or fraternity has already passed the verdict of inefficiency upon himself, in the court of his own mind, as certainly as that injustice to the world holds the dominion in the mind of him who is unwilling thus to treat each member of the race, community, or profession.

Instead of majorities of professional bodies usually being in the right in matters of dispute, it is the rule for them to vote against any doctrine or proposition progressive in character.

It has been considered a justifiable "trick of the trade" in lawyers to study the bearings of law and court decisions for the express purpose of evading them, or rendering nugatory their provisions.

Physiologists and pathologists, on the contrary, study the laws of the economy in health and disease for purposes of obedience in their own persons and those committed to their charge.

The best possible cure for egotism is extended information:—As the best antidote to professional bickerings is free, fraternal and intimate frequent intercourse.

It is often said "I have no time to observe the requirements of professional etiquette," when the truth is, God never gives time for anything else in a professional capacity.

Could we be wholly engaged in seeing how much good we can effect in our calling instead of how soon we can acquire the means to give up the exercise of our powers in their legitimate sphere; we should soon cease to hear of this one and that one being such a nuisance in the profession or the community.

A few examples of whole hearted men will go far to point the way in which we ought to go; and such is the spontaneous tendency of human minds that they will pursue the right so soon as there is no longer any doubt in which direction she holds her court.

All men, ignorant and learned, are in pursuit of that which seems good to them, and for the time each one does honestly pursue it; too many, however, are in search of immediate in contradistinction to prospective good.

In the good to be realized from observance of etiquette, we are like the agriculturist, we must sow good seed in the dark and lowering day, in hopes to harvest in the bright sunshine of future joy.

The lot of the sower of good seed is not a miserable one by any means, for while he confides his precious burthen to the soil at morning and evening, he has the blessed assurance that at least some of it will bring forth many fold more than it were possible for it to do were it still kept high and dry in his private garner, out of sight, and out of the reach of the blessedness of a generous use.

But he who sows the tares of discord, and in harmony of isolation, seclusion, and selfishness, is by no means a frank, bold, or happy man, for he is self-condemned already, and sneaks away in the dark to do his unholy work, lest he should be known and despised by the community, as justly as he already has himself dispised, the meanness of the act that can alone gratify a base born nature, which is its own perpetual scourge, lashing its hapless possessor through the mazes of an unsatisfied life.

Be assured, my beloved brethren, that none of us can afford to consume time in criminating any but our own dear selves at the bar of an enlightened conscience.

If we are busy, as we ought to be, correcting our own errors, it will be apparent in our lives and conduct, and prove much more potent than all the fine spun arguments against our fellows.

The best possible bar to which to bring a culprit is that of his own conscience, because he can not escape the decisions of that court by running out of its jurisdiction.

And the best way to obliterate the memory of past errors and sins in our career is to be so busy at improvement as to pre-occupy our minds most of the time therewith, and thus, at least, secure partial immunity from the painful reflections.

These pains will keep us in a good mood to tenderly deal with the lapses of our fellows when we must encounter them, and teach us how grateful a boon it is to be mercifully dealt with by others.

I am then in favor of the so called "unwritten code," which is the only efficiently written and always ready talisman of each individual act of professional or social life.

That which has become a persistent presence with us needs no prompting to remember, nor deep erudition to define and explain.

As certainly as that there will always be occupants for jails and houses of correction where these are erected and maintained, will we find infractors of etiquette where this is enacted for the protection and benefit of a special class.

To him who is thoroughly grounded in upright and holy thought, word, and deed, the enactment and maintainance of the formality of law and etiquette are neither a "terror" nor delight, for he is habitually far above the need of any such external restraint or guide.

Until we cease to oppose littleness, selfishness, exclusiveness, and meanness with their own methods of procedure, we shall utterly fail to fulfil the mission committed to our hands of bringing in the better times so long promised.

Does any say this is all well enough theoretically, but practically it is maintainable? Let it be remembered that all practicalities are first conceived in theory, and through much of labor, and may be repeated blunders and failures, at last becoming established as practical reality, after which they are imbibed without other than the labor of imitation, and are no longer regarded as but "fanciful conceptions of some hairbrained visionary;" but the sober realities of life, which not one in ten could, if asked, trace to their origin and establishment.

He who looks out of himself for discriminations of what is and what is not in accordance with professional etiquette, will ever be in doubt which way to decide impartially any question pertaining hereto brought before the court of his mind. While he who is a whole man will never be at a loss to settle at once what is or is not a correct procedure toward others of the profession or of the world, simply because he is always ready to cordially observe the unsophisticated practice of the *Golden Rule.*

